# Assessing the future threat from vivax malaria in the United Kingdom using two markedly different modelling approaches

**DOI:** 10.1186/1475-2875-9-70

**Published:** 2010-03-05

**Authors:** Steven W Lindsay, David G Hole, Robert A Hutchinson, Shane A Richards, Stephen G Willis

**Affiliations:** 1 Disease Control and Vector Biology Unit, London School of Hygiene and Tropical Medicine, Keppel Street, London WC1E 7HT, UK; 2 Center for Applied Biodiversity Science, Conservation International, 2011 Crystal Drive, Suite 500, Arlington, VA 22202, USA; 3 School of Biological and Biomedical Sciences, Durham University, South Road, Durham DH1 3LE, UK

## Abstract

**Background:**

The world is facing an increased threat from new and emerging diseases, and there is concern that climate change will expand areas suitable for transmission of vector borne diseases. The likelihood of vivax malaria returning to the UK was explored using two markedly different modelling approaches. First, a simple temperature-dependent, process-based model of malaria growth transmitted by *Anopheles atroparvus*, the historical vector of malaria in the UK. Second, a statistical model using logistic-regression was used to predict historical malaria incidence between 1917 and 1918 in the UK, based on environmental and demographic data. Using findings from these models and saltmarsh distributions, future risk maps for malaria in the UK were produced based on UKCIP02 climate change scenarios.

**Results:**

The process-based model of climate suitability showed good correspondence with historical records of malaria cases. An analysis of the statistical models showed that mean temperature of the warmest month of the year was the major factor explaining the distribution of malaria, further supporting the use of the temperature-driven processed-based model. The risk maps indicate that large areas of central and southern England could support malaria transmission today and could increase in extent in the future. Confidence in these predictions is increased by the concordance between the processed-based and statistical models.

**Conclusion:**

Although the future climate in the UK is favourable for the transmission of vivax malaria, the future risk of locally transmitted malaria is considered low because of low vector biting rates and the low probability of vectors feeding on a malaria-infected person.

## Background

Over the last 30 years there has been a rapid increase in emerging and re-emerging infectious diseases in the human population [1-3], with over 175 species of pathogens now classified as emerging or re-emerging [4]. Emergence of these diseases are frequently associated with ecological changes [1] and many are transmitted by insect or tick vectors [1,4,5]. Because of the sensitivity of malaria to a range of environmental factors there is concern that the disease may spread to new regions of the world [6,7], previously disease free, like the UK.

Malaria was once common in marshland communities in central and southern England between 1500 and 1800, before finally disappearing in the early 1900s [8]. Those areas most badly affected included the Fens, Thames estuary, the Kent coast, the Somerset levels, the Severn Estuary and the Holderness of Yorkshire; all substantial areas of marshland. The disease declined progressively from the 1850s onwards, as living conditions in the marshes improved and quinine, an effective anti-malarial, became more affordable and was used more frequently. The last significant epidemic of malaria in England and Wales occurred in 1917 and 1918, with the main focus on the Isle of Sheppey, near the mouth of the Thames Estuary [9,10]. During this epidemic, there were 330 cases of locally-transmitted vivax malaria when infected servicemen returning from Macedonia were billeted near salt marshes. Indeed, all reported cases of locally transmitted malaria in the 1900s were vivax malaria, except for an unusual case of falciparum malaria in Liverpool [11]. Whilst vivax malaria is a serious and debilitating infection, it is rarely a killer like falciparum malaria [12]. Thus although historically mortality rates in the marshlands of southern England were several orders higher than inland areas, it is unlikely that the elevated mortality in the marshes was due to vivax malaria.

There are six species of Anophelines in Britain capable of transmitting both temperate and tropical strains of vivax malaria: *Anopheles atroparvus, Anopheles algeriensis*, *Anopheles claviger*, *Anopheles daciae*, *Anopheles messeae *and *Anopheles plumbeus *[13,14]. *Anopheles atroparvus *is considered the most important vector of vivax malaria in the UK, since its distribution coincides best with the historical distribution of the disease [15]. It also rests inside homes and will feed on people [16].

Modelling the threat from malaria is a necessary first step in estimating the populations at risk and where they live [17-19], helping to inform disease surveillance programmes across the world. To date there have been two broad approaches to modelling the distribution of malaria: process-based models and statistical ones. Process-based models of malaria spread are considered favourable since they are based on an understanding of the biology of malaria. They are largely driven by the manner in which temperature influences mosquito survival, frequency of blood-feeding and the development of parasites within mosquitoes [20,21]. The major concern with this approach is that the models are solely temperature driven and no allowance is made for two potentially important environmental parameters known to affect vector populations; rainfall and relative humidity. Rainwater, groundwater and diluted seawater provide mosquito breeding habitats, along with a moderately humid environment which is conducive to vector survival [22]. Statistical models capture the climate envelope of where the disease is present and produce relatively robust models of the disease's current distribution [18,19]. However, they lack the clarity and understanding provided by process-driven models. Rarely are the two different modelling approaches used together. A simple process-driven model of malaria was compared with a statistical model to examine the risk of malaria transmission in the UK today and in the future under projected climate change.

## Methods

### The process-based approach

The primary analyses were based on the classic concept of the basic reproduction rate (*R*_0_) [23,24], which represents the number of future cases of malaria derived from one infective case at the present time, before this case is cured, or the infected person dies. This temperature-driven model follows on from earlier work [21]. One common expression for *R*_0 _is shown below:

where, the product *ma *is the expected number of mosquito bites per person per day (or equivalently, per night). It was assumed that this expectation equalled 1, since we thought that few people would tolerate more than one bite each night. The feeding rate (bites/person/day) is calculated using:

where *h *is the proportion of mosquito blood meals taken from people (rather than animals that are not infected with human malaria) and *u *is the period in days of the gonotrophic cycle - the interval between laying each egg-batch and, generally, each mosquito blood meal. The present model assumes a mean value of *h *of 0.42 for indoor-resting mosquitoes [16]. The length of the gonotrophic cycle is calculated using:

where *f*_1 _is a thermal sum, measured in degree days, that represents the accumulation of temperature units over time to complete the cycle (36.5°C), *g*_1 _is a threshold below which development ceases (9.9°C), and *T *is ambient temperature [25]. *p *is the daily survival probability of adult mosquitoes. The present model takes the median value of the mortality rate for *An. atroparvus *= 0.029/day (n = 24, range 0-0.294/day) [16]. *n *is the period of parasite development in adult mosquitoes, in days (the sporogonic cycle) and is given by:

where *f*_2 _is a thermal sum, measured in degree days, representing the accumulation of temperature units over time to complete the development (105 degree days), *g*_2 _is a development threshold below which development ceases (14.5°C), and *T *is ambient temperature [16]. Generally, as conditions warm the rate of parasite development increases [26,27]. However, there is uncertainty about the minimum threshold for parasite development, with figures ranging from 14.5-16.0°C [25,26,28]. *b *is the proportion of female mosquitoes developing parasites after taking an infective blood meal (0.19) [29]. *r *is the rate at humans with malaria recover from the infection. It is usually considered that the duration of each infection is therefore 1/*r *days. It was assumed that an average infection would be patent for 60 days, giving a value for *r *of 0.0167/day [26].

In the model, these formulae were combined with the 1961-90 UKCIP climate data at a 5 km resolution for present-day climate. The model outputs the number of months of the year when *R*_0 _is >1, indicating potential disease spread. Under conditions when *R*_0 _is <1 for a considerable proportion of the year, the disease probably cannot persist without continuous introduction from elsewhere, or possibly as quiescent stages within apparently recovered people. Future risk was modelled for 2015 and 2030 using the UKCIP02 Medium-high climate change scenario for the UK. This scenario uses the Hadley Centre global climate model (HADCM3) for a medium-high climate change scenario (SRES A2), which is used, via a further stage, to drive a regional version of the model that has a resolution of 50 km over Europe. Results from this regional model over the UK then form the UKCIP02 scenario. The distribution of saltmarsh was derived from the Land Cover Map of Great Britain 1990 [30] which classifies land use in the UK into 25 classes at a 25 m resolution from satellite information. 1 km cells were classified as containing saltmarsh if they contained more than 0.5% saltmarsh according to the Landcover map. To validate this model, climate suitability maps showing the potential distribution of malaria in 1961-1990 were compared with distribution records of malaria recorded in 1859 and 1864 [31]. To demonstrate whether temperatures were comparable between these periods, annual summer temperatures (June to September) from the Central England Temperature record [32] were compared for 1831-1860 and 1961-1990 using a t-test with SPSS software (PASW Statistics 18).

### The statistical approach

**Figure 1:**
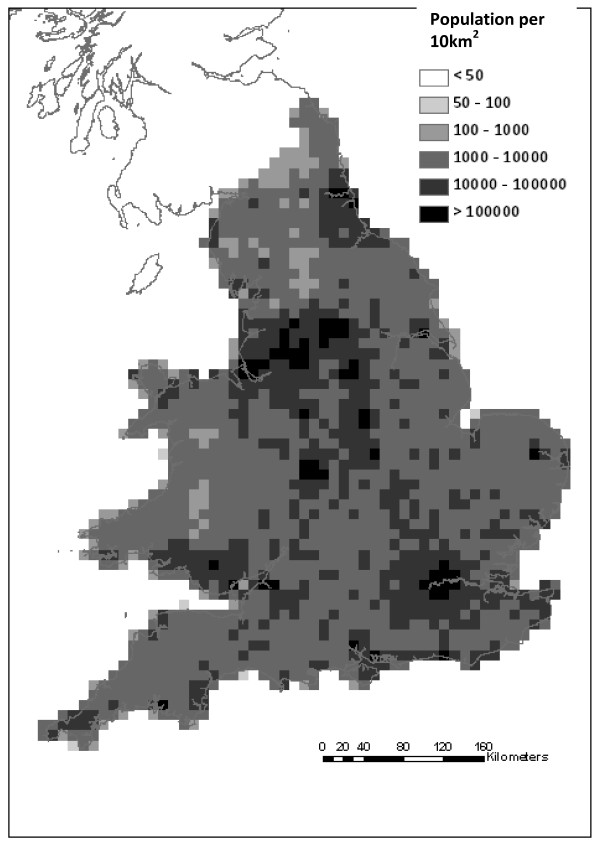
**Population density in England and Wales in 1911**.

Logistic regression was used to look for evidence that environmental conditions and population density were informative for predicting the likelihood of locally-contracted cases of vivax malaria being reported during 1917-18 [9]. First, the UK was sectioned into 10 km × 10 km grid cells. For each cell, three biologically relevant environmental factors were considered: the mean temperature of the warmest month (MTW), mean temperature of the coldest month (MTC), and an index of wetness (APET) which is a measure of potential evaporation in relation to rainfall. Population density for each cell was estimated using parish population data from the 1911 census in England and Wales [33]. Area of parish was recorded in acres and mapped to a grid of parish boundaries for England and Wales in 1911 [34]. The resulting population map was then re-sampled at the 10 km × 10 km scale (Figure 1). Given the large range in densities, the natural logarithm of population density (LnP) was considered as a predictor variable. The number of malaria cases imported as a result of infected returning servicemen (Imp) was also recorded for each cell [9], since it was considered that local infections would be more likely when an infected soldier returned home.

Let *p *denote the probability that a cell will contract at least one case of vivax malaria during 1917-1918. It was assumed *p *was logistic:

where the *x*_*i *_are the measured factors associated with the cell and *β*_*i *_are the model parameters to be fit to the data. Models were constructed by setting various combinations of the *β*_*i *_to zero, and, in all, 32 models were considered, changing the combination of biological and human factors (i.e. MTW, MTC, LnP and Imp) assumed to be informative. For this analysis, the log-likelihood of the data, given model M, was calculated using:

where *θ*_*M *_is the set of non-zero parameters that define model M, and *x *is the set of factors associated with cell *i*. Here, it was assumed that the 1,618 cells, for which we have data, are independent. Given the scarcity of locally-contracted malaria (only 23 recorded cases), more complex spatially-explicit models that incorporate non-independence was not considered in the analysis, as there was unlikely to be sufficient information in the data to support such complexity.

In order to determine which of the 32 models best described the data, model selection using Akaike's Information Criterion (AIC) was employed as a metric of model parsimony [35]. AIC seeks a trade-off between model fit and model complexity. Complex models, having numerous parameters, often fit the data well, but are associated with increased uncertainty in their parameter estimates. AIC is a technique for identifying models having complexity that can be supported by the data. Each model is associated with an AIC value, calculated using:

where *M *denotes the model and *θ *is the set of *K *model parameters that are estimated from the data using maximum likelihood. Once AIC-values had been calculated for all models a Δ-value was calculated for each model, which is the difference between the AIC value of the model and the lowest AIC value calculated among all models. Thus, the model with the lowest AIC value was associated with a Δ-value of zero. Models considered to be parsimonious with the data were those that had a Δ-value < 6 [36]. In addition, to avoid selecting overly complex models, we also disregarded models if they were more complex versions of models having a lower Δ-value [36].

## Results

### Processed-based model

**Figure 2:**
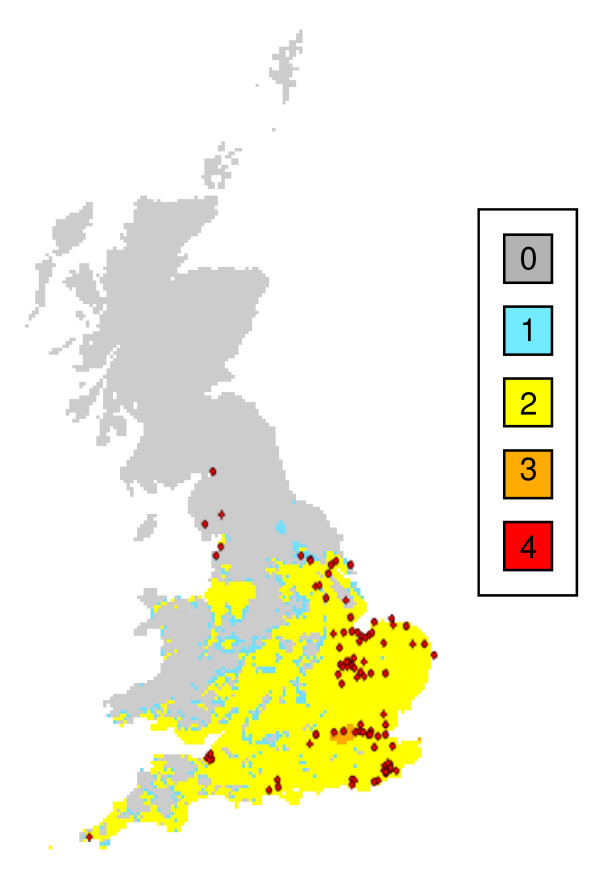
**Malaria risk across Great Britain for the 1961-1990**. Shading represents the number of months where the climate could support vivax malaria if it were introduced. Red circles show cases of ague (some of which will have been malaria cases) in the 19^th ^Century [31].

**Figure 3:**
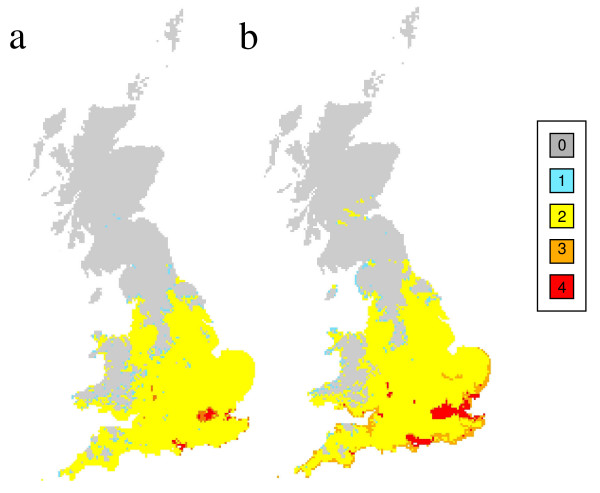
**Malaria risk across Great Britain for 2015 (a) and 2030 (b)**. Shading represents the number of months where the climate could support vivax malaria if it were introduced.

**Figure 4:**
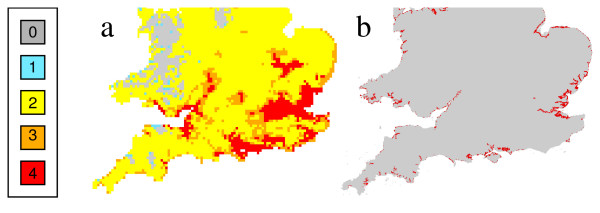
**Climate suitability zone for vivax malaria in the southern UK in 2030 (a) and areas of saltmarsh in 1990 (b)**. Shading represents the number of months where the climate could support vivax malaria if it were introduced.

Maps of malaria suitability using present day climate (Figure 2) and for the two future periods (Figures 3a and 3b) show the number of months that vivax malaria, if it were introduced, could persist each year in different parts of the country. The 1961-1990 distribution of malaria risk (Figure 2) corresponded extremely well with past records of ague cases (that are likely to have included patients with malaria) in England during the nineteenth century [31], with 93 of the 96 cases of ague situated within the area deemed suitable for malaria. Summer temperatures were similar in 1831-1860 (mean = 14.7°C, 95% Confidence intervals, CI = 14.4-15.0°C) and 1961-1990 (mean = 14.9°C, 95% CIs = 14.6-15.2°C, t test = -1.135, p = 0.26). Future scenarios for 2015 and 2030 demonstrate that the climate suitable for vivax transmission could persist in large parts of central and southern England for two months each year. Areas where the climate would be suitable for three to four months each year, include London, the coastline of south and south-eastern England, the mouth of the Severn Estuary and the Fens. Although the climate is suitable for vivax transmission in large areas of the UK, within this area the saltmarsh habitat for *An. atroparvus *is confined to relatively narrow strips of coastline (Figures 4a and 4b).

### Statistical models

**Figure 5:**
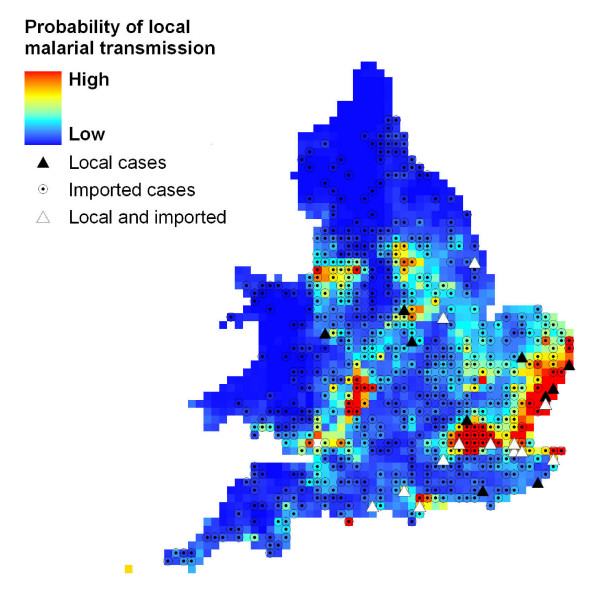
**Maximum-likelihood based model indicating probability of malarial occurrence**. Solid triangles represent locations of locally-contracted cases of malaria in 1917/18 [9].

Further analysis with our statistical model, generated using an independent data set, showed that the resulting climate suitability map (Figure 5) was similar to that generated by the process-based model (Figure 3). The AIC analysis strongly supported mean temperature during the warmest month (MTW) as an important predictor variable of locally contracted malaria during 1917-18 (Table 1). The analysis also suggested weaker support for soil moisture and population density, and these factors were only informative once MTW was accounted for. The best AIC model was M(MTW + APET + LnP), and was parameterised by logit(*p*) = -21.5+1.91*x*_MTW_-16.4*x*_APET_+0.30*x*_LnP_. Thus, malaria was more likely under warmer conditions, when population density was higher and in drier conditions.

**Table 1:** Model selection results from the AIC analysis investigating the importance of environmental and population data on the prevalence of new malaria cases between 1917 and 1918.

Model factors	*K*	AIC-value	Δ-value
MTW	2	213.2	3.7

MTW + APET	3	210.6	1.1

MTW + APET + LnP	4	209.5	0

Presented are those models, of the 32 considered, that (1) had a Δ-value < 6 (defined as the AIC-value of the model minus the smallest of all models) and (2) were not more complicated versions of models having a lower AIC-value. Model factors are abbreviated as: mean temperature during the warmest month (MTW), wetness (APET), and log population density (LnP).

## Discussion

The simple temperature-driven processed-based model demonstrated that present temperatures in the south of England are able to support transmission of vivax malaria for a few months each year. For most areas, this is just for two months, although the heat island effect of London means this city could support transmission for three months. If the climate becomes warmer, conditions for transmission become more favourable and last for longer. Under the UKCIP02 medium-high scenario used here, the risk of transmission is predicted to increase in the south of England, spreading northwards towards the Scottish border. The areas at greatest risk include the Thames Estuary, the Suffolk coast, the Fens, Romney marshes, the Southampton coastline, the Severn Estuary and parts of the South-West coastline. Interestingly, by 2030, the areas where the climate is predicted to be suitable for malaria for three to four months are those that once supported malaria in the past: coastal and inland marshes of southern England. It is important to appreciate that this analysis reflects average monthly values, and that in reality there will be large variations in climate from year to year, making some years considerably more suitable for malaria transmission than others.

It is likely that our predictions are robust for two reasons. Firstly, 93 of the 96 sites where malaria was recorded in the early-nineteenth century occurred within the climate suitability map for malaria in 1961-90, as reported previously [37]. Although the cases and climate surfaces were from different periods, the summer temperatures, when conditions are best for transmission, were similar. Secondly the climate suitability map generated using a statistical model, fitted to an independent data set, was very similar to that generated using the process-based model.

An analysis of the statistical models demonstrated that the most important parameter to include was the mean temperature of the warmest month, providing support for using the temperature-driven process-based model in the analysis. Risk of malaria also increased with higher human population densities and drier conditions. Whilst it is logical to appreciate that the risk of an infective mosquito feeding on a person will be greater when there are more people, it is perhaps surprising that malaria was predicted to be more likely in drier conditions. This unexpected result was largely due to five cases of malaria occurring around the Suffolk coast that could not be accounted for by warm temperatures or high population density. However, since this region is close to saltmarsh, the favoured habitat of the historical malaria vector, *An. atroparvus*, it may simply reflect that the cases lived close to suitable vector habitats. This raises a general problem with our mapping approach. And that is, although climate governs the distribution of vectors and the pathogens they transmit at a broad scale, the actual risk depends on local conditions (e.g. habitat type) and the complex interactions between pathogen, vector and people.

Within the temperature-suitability zone we are uncertain where the vectors occur. The actual distribution of potential vectors in the UK is poorly defined and is based on data collected over 100 years ago or by entomologists in an unsystematic fashion [31,38-40]. The concern is that these distributions may not reflect the true distribution of vector species. As a proxy measure to identify sites likely to have *An. atroparvus*, a landcover map of saltmarsh areas was used as a filter over the temperature-suitability map, since this mosquito is a coastal species, tolerant of brackish water [41].

At first sight these maps may give the impression that there is a significant risk of malaria returning to the UK. However, the field studies cast doubt on this conclusion and demonstrate the importance of carrying out fieldwork to validate the model predictions. In 2003, a survey of adult mosquitoes was carried out on the saltmarsh on the Isle of Sheppey [42], the last place in England to experience an epidemic of malaria [9]. Using two carbon dioxide baited CDC light traps and two Mosquito Magnet^® ^traps mosquitoes were sampled continuously from June to September, the period when most mosquitoes are searching for a host. At 1240 ha this is the largest area of saltmarsh in the UK/England, yet only 40 *An. maculipennis *(probably *An. atroparvus*) were collected. Of 83 blood-fed *An. maculipennis *collected from a horse stable, a derelict school and a derelict concrete pill box on the Isle of Sheppey (RH unpublished data), and analysed using a precipitin test [43], just two had fed on people indicating that around 2% of the population fed on people. The risk of being bitten by a potential malaria mosquito is, therefore, extremely low, even though there is a general problem with nuisance biting by other mosquito species on the Island [42].

How likely is it that vivax malaria could be transmitted in the UK? Malaria transmission requires that a potential vector should feed on someone carrying gametocytes, the stage of the parasite that is capable of maturing and becoming infective within a mosquito. Between 9-24 days after taking an infectious blood meal of gametocytes, depending on temperature [26] a vector mosquito will be potentially able to transmit the infection to anyone that it bites. In 2003 there were 206 cases of imported vivax malaria and approximately 66% of these were contracted by visitors to the Indian subcontinent [44]. It is likely that this will under-represent the true number of people with circulating gametocytes since even in areas of low transmission many infectious semi-immune patients will not appear sick [45]. Nonetheless, overall there are likely to be relatively few people carrying vivax gametocytes in the country, and since most of these are amongst people of Asian decent who tend to live in major urban areas, away from coastal marshes, the possibility of *An. atroparvus*, biting an infectious patient are remote. There remains the possibility that there are other vectors of vivax malaria in the UK since not all indigenous cases of malaria recorded in the 1917/18 outbreak lived near saltmarsh. In such cases, *An. plumbeus *or *An. claviger *might have transmitted the infection.

Overall, it is seems extremely unlikely that vivax malaria will be locally transmitted within the UK. Therefore, not more than a few very rare cases of autochthonous malaria in the UK would be expected over the next 50 years. Indeed, one is much more likely to be struck by lightning [46] than to get malaria from an English mosquito.

The threat posed by malaria in Europe has been assessed previously [47-49]. Processed-based [50] models and statistical ones [19] have both been used to explore the global risk of malaria, whilst at the local and countrywide scales processed-based models, of differing levels of complexity, have been used for assessing future risk in Europe (France [51,52], Germany [53] and Portugal [54]). The present study differs from these since it uses both processed-based and statistical models to explore the risk of malaria, and validates both models with independent data sets of historical cases of malaria.

## Conclusion

In conclusion, predicting disease risk based solely on climate-driven models is a logical and useful first step in the process of predicting risk. In the case of vivax malaria transmitted by *An. atroparvus *in the UK, more local factors become important, including the presence of specific habitats for mosquitoes and the distribution and abundance of infectious patients. The important message is that considerably more field-based research should be undertaken to test and improve the predictions made by the relatively simple models shown here.

## Competing interests

The authors declare that they have no competing interests.

## Authors' contributions

SWL conceived the study and developed the process-based model. SAR and SGW did the statistical analysis and SGW and DGH carried out the spatial analysis. RAH carried out the fieldwork. SWL and SGW wrote the manuscript, and all authors contributed to the drafts and read and approved the final manuscript.
